# MyoLoop: Design, development and validation of a standalone bioreactor for pathophysiological electromechanical in vitro cardiac studies

**DOI:** 10.1113/EP091247

**Published:** 2023-10-17

**Authors:** Fotios G. Pitoulis, Jacob J. Smith, Blanca Pamias‐Lopez, Pieter P. de Tombe, Danika Hayman, Cesare M. Terracciano

**Affiliations:** ^1^ National Heart & Lung Institute Imperial College London London UK; ^2^ Department of Physiology and Biophysics University of Illinois at Chicago Chicago Illinois USA; ^3^ Laboratoire “Physiologie Et Médecine Expérimentale du Coeur Et Des Muscles,” PhymedexpINSERM, CNRS Montpellier University, CHU Arnaud de Villeneuve Montpellier France

**Keywords:** cardiac cycle, electromechanical stimulation, in vitro culture, living myocardial slices, mechanical load, MyoLoop

## Abstract

Mechanical load is one of the main determinants of cardiac structure and function. Mechanical load is studied in vitro using cardiac preparations together with loading protocols (e.g., auxotonic, isometric). However, such studies are often limited by reductionist models and poorly simulated mechanical load profiles. This hinders the physiological relevance of findings. Living myocardial slices have been used to study load in vitro. Living myocardial slices (LMS) are 300‐μm‐thick intact organotypic preparations obtained from explanted animal or human hearts. They have preserved cellular populations and the functional, structural, metabolic and molecular profile of the tissue from which they are prepared. Using a three‐element Windkessel (3EWK) model we previously showed that LMSs can be cultured while performing cardiac work loops with different preload and afterload. Under such conditions, LMSs remodel as a function of the mechanical load applied to them (physiological load, pressure or volume overload). These studies were conducted in commercially available length actuators that had to be extensively modified for culture experiments. In this paper, we demonstrate the design, development and validation of a novel device, MyoLoop. MyoLoop is a bioreactor that can pace, thermoregulate, acquire and process data, and chronically load LMSs and other cardiac tissues in vitro. In MyoLoop, load is parametrised using a 3EWK model, which can be used to recreate physiological and pathological work loops and the remodelling response to these. We believe MyoLoop is the next frontier in basic cardiovascular research enabling reductionist but physiologically relevant in vitro mechanical studies.

## Abstract (highlights)

- **What is the central question of this study?**
  Can cardiac tissues be cultured under conditions that do not simulate the mechanical load the heart is exposed to in vivo?
- **What is the main finding and its importance?**
  A culture bioreactor was developed that can keep living heart tissue alive in vitro under continuous electromechanical stimulation. The device, MyoLoop, simulates the cardiac cycle on a beating slice during culture, recreating the in vivo workload of the heart in vitro. MyoLoop is a novel translational platform for cardiac studies.

## 1 INTRODUCTION

Cardiovascular disease remains the biggest cause of disease burden globally with major associated healthcare costs (Savarese & Lund, 2017). In the United States alone medical costs of cardiovascular diseases (CVD) including heart failure, coronary heart disease, hypertension and stroke, are expected to reach $818 billion per year by 2030 (Reuters, 2012). Translational research is critical for the development of new therapeutics that can reduce the burden of CVD; however, to this date, the promise of new therapies has remained unfulfilled. Of the U.S. Food and Drug Administration (FDA) drugs approved within the last 5 years, only six out of 248 were for CVD, despite CVD accounting for one‐quarter of deaths in the USA alone. There are many reasons for this gap, yet here we focus on one that starts early in the therapeutic pipeline: the failure of many results obtained in preclinical in vitro cardiac models to translate to the bedside. We present a new translational cardiac platform for preclinical studies, MyoLoop, and discuss its role in this evolving field.

in vitro cardiac preparations are indispensable tools in basic cardiovascular research. They range from isolated cardiomyocytes to papillary muscles and engineered heart tissues, and more recently living myocardial slices. in vitro models are more reductionist than their in vivo counterparts as the effects of multiple interacting systems present in vivo can be controlled with higher confidence in vitro (Pitoulis et al., 2019). Though this allows greater degrees of causality between dependent and independent variables, their oversimplified nature means that in vitro models often do not recapitulate essential properties of the in vivo heart (Fang & Casadevall, 2010; Kofron & Mende, 2017).

Our lab has long advocated the development of more physiologically relevant cardiac models (Nunez‐Toldra et al., 2022; Perbellini et al., 2018; Pitoulis et al., 2019, 2021; Watson et al., 2019), which are able to simulate both the structure and the function of the adult heart. Over the past decade, we have described and validated a new cardiac model, the living myocardial slice (LMS). LMSs are 300‐μm‐thick living cardiac tissues prepared from the hearts of small and large mammals, including human hearts, using a high‐precision vibratome (Brandenburger et al., 2012; Perbellini et al., 2018; Watson et al., 2017). Because LMSs are prepared directly from cardiac tissue, their structure (extracellular matrix (ECM), cell–cell interactions, cell heterogeneity) and function (e.g., contractility, electrophysiology) fully reflect the properties of the tissue from which they are obtained. Importantly, we have previously estimated that approximately only 3% of all cells within a LMS are dead following a vibratome preparation. This makes them highly physiologically relevant and has been exploited by the cardiac community to interrogate metabolism, electrophysiology, mechanics and molecular and genetic signatures of both healthy and diseased tissue alike (Brandenburger et al., 2012; Camelliti et al., 2011; Fischer et al., 2019; Nunez‐Toldra et al., 2022; Perbellini et al., 2017, 2018; Pitoulis et al., 2020).

In addition to a model approximating the structure of the in vivo tissue, the function of the heart must be adequately simulated for the model to be physiologically relevant. Recreating the mechanical load that the heart is experiencing is essential for this. The principal operation of the heart is to pump blood into the circulation. Although there are many variables that affect the mechanical axis, approximating the in vivo mechanical function necessitates at minimum simulation of preload and afterload. Preload is the end‐diastolic pressure of the myocardium prior to electrical depolarisation and the onset of contraction; it maintains the steady‐state of the closed‐loop vascular system by linking venous return to cardiac output. Afterload is the load against which the heart needs to contract to eject blood into the vasculature during systole. Preload and afterload can affect both the short‐ and long‐term operation of the heart, as seen with differential remodelling that occurs with increased preload (e.g., aortic stenosis, pressure overload) or afterload alike (e.g., mitral valve regurgitation, volume overload; Pitoulis et al., 2021; Toischer et al., 2010).

**FIGURE 1:**
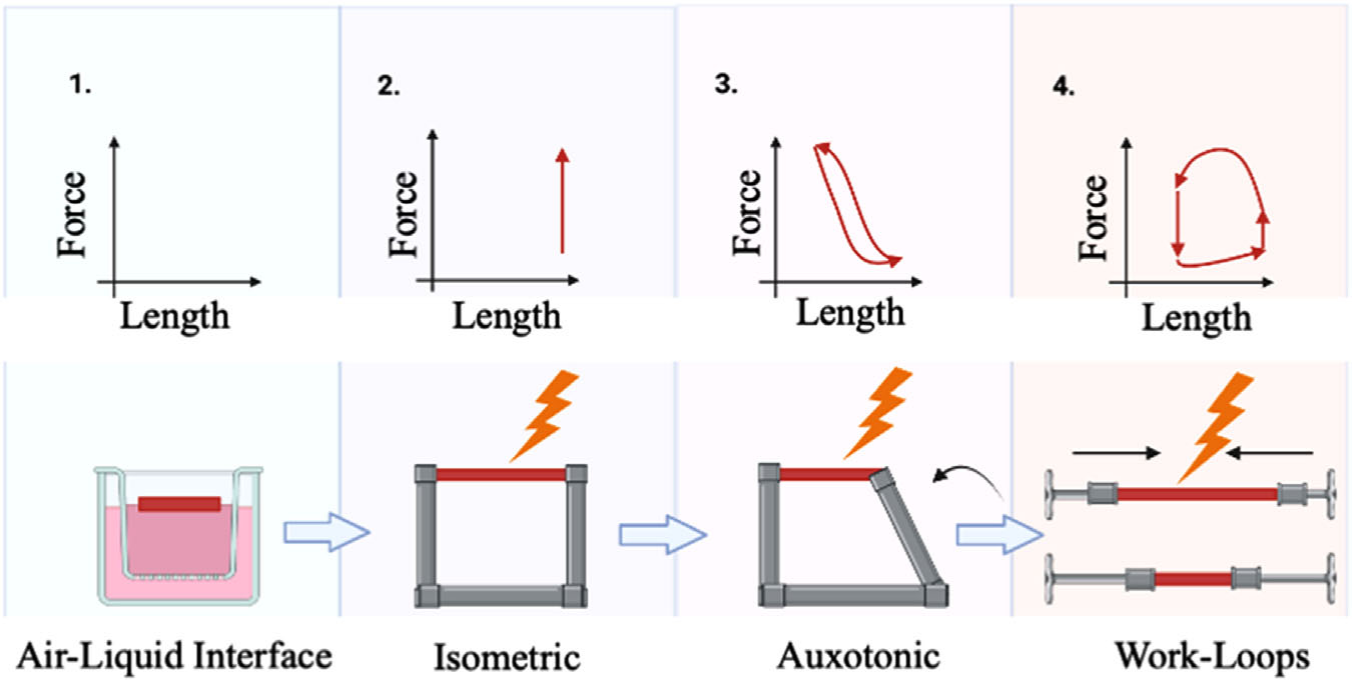
Progression of cardiac slice culture platforms. Over the past decade, multiple different platforms to culture LMSs have been developed. Starting with the air–liquid interface, where LMSs were kept unloaded to isometric load on inflexible posts, to auxotonic load using flexible posts, and more recently using length actuators to recreate the cardiac cycle. The differences in mechanics of each of these approaches can be appreciated on the force–length plane.

Given the importance of the mechanical axis, multiple protocols have been developed to culture LMSs under electromechanical stimulation (Brandenburger et al., 2012; Fischer et al., 2019; Pitoulis et al., 2021; Watson et al., 2019). Such protocols mimic mechanical load and enable LMSs to be maintained at a steady‐state that more closely mimics the in vivo environment, permitting temporal relationships between interventions and phenotype to be studied reliably. Over the span of almost two decades, the culture methodology of LMSs has gone through a series of incremental optimisations, starting with culture on air–liquid interface (Brandenburger et al., 2012) through to electromechanical stimulation protocols under paced‐isometric load (Watson et al., 2019), paced‐auxotonic load (Fischer et al., 2019) and more recently paced‐work loops (Pitoulis et al., 2021) (Figure 1). Each iteration has approximated the real‐life operation of the heart more closely, and thus our ability to maintain LMSs in vitro and study cardiac pathophysiology reliably.

Brandenburger et al. (2012) suspended LMSs in an air–liquid interface, in a technique considered at the time to be the gold standard for culture. Although LMSs were viable after 28 days, the absence of mechanical and electrical stimulation led to significant cardiac remodelling including loss of contractility as well as essential cardiomyocyte structural elements (Brandenburger et al., 2012). The next culture method originated from our lab when Watson et al. (2019) used culture chambers with stretchers onto which the LMS could be mounted. The stretchers contained stainless steel posts, which could be adjusted in length allowing control of LMS preload. As the posts were inflexible, upon stimulation LMSs contracted isometrically (Figure 1). The importance of mechanical load application was highlighted by Perbellini et al. (2018) who showed that the application of mechanical load prevents proliferation of cardiac fibroblasts in LMSs. Although this approach was a significant step forward as it allowed approximation of preload together with pacing, manipulation of afterload was absent and LMSs were beating under unphysiological isometric load (Watson et al., 2019). A similar system was developed by Fischer et al. (2019), in which LMSs were mounted on flexible posts and stimulated to contract auxotonically. In contrast to the system by Watson et al. (2019), this allowed a variable afterload, albeit one that did not accurately reproduce the afterload experienced by the heart in vivo (Figure 1).

Recently, we described a culture method that can recreate the pressure–volume loops experienced by the heart in vivo as force–length work loops on LMSs (Pitoulis et al., 2021). To do that, we used a three‐element Windkessel (3EWK) model to electromechanically load the LMSs, applying both preload and afterload to the tissue under pacing conditions. Specifically, preload was applied by stretching the LMS to the desired sarcomere length (SL), while afterload was applied by using the 3EWK parameters: arterial impedance (Ra), compliance (Ca) and peripheral resistance (Rc). The dynamic mechanical events of the in vivo cardiac cycle with the distinct phases of isometric contraction, ejection, isometric relaxation and diastolic refilling were thus simulated on LMSs and applied continuously over a chronic 3‐day culture (Figure 1).

**FIGURE 2:**
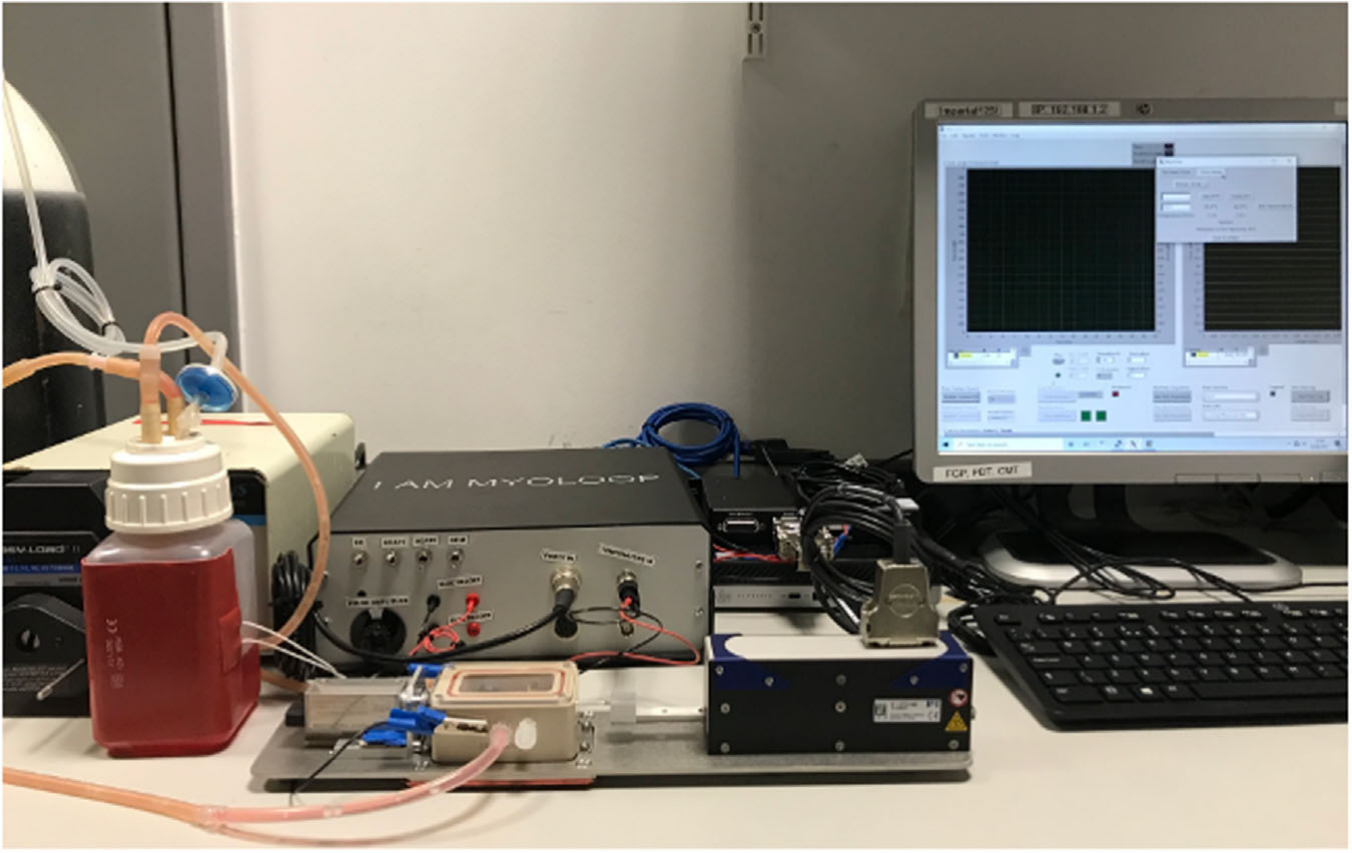
MyoLoop, a benchtop bioreactor. MyoLoop consists of a physical assembly, which includes the culture chamber where the LMSs are maintained, the force and length actuator, the instrumentation box (seen at the back), as well as the custom software that runs the 3EWK model and data acquisition, stimulation and thermoregulation modules (seen on the screen on the right).

This culture method was the first time that work‐loops using a 3EWK model were applied chronically during in vitro LMS culture. However, the proof‐of‐concept platform demonstrated in Pitoulis et al. (2021) was developed by extensively modifying commercially available instruments that were not suited for prolonged culture experiments. As a result, the platform previously described in Pitoulis et al. (2021) was (a) low‐throughput, (b) non‐modular, (c) non‐scalable, (d) not user‐friendly, (e) not readily customisable and (f) very expensive to develop. To address these limitations, we set out to develop a user‐friendly self‐contained benchtop dynamic biomimetic cardiac culture system, MyoLoop (Figure 2). We herein describe the design, development and validation of MyoLoop, a commercially available bioreactor that can be used to culture cardiac preparations in vitro under user‐defined pathophysiological electromechanical stimulation.

## 2 METHODS

### 2.1 Ethical approval

All animal experiments were conducted in accordance with institutional and national regulations, and approved by Imperial College London, under license by the UK Home Office, UK Animals (Scientific Procedures) Act 1986 Amendment Regulations 2012, and EU directive 2010/63/EU (ref no.: 09/H0504/104+5 CBRU Biobank).

### 2.2 MyoLoop overview

MyoLoop is divided into four main systems and each of these is divided into subsystems. Systems and subsystems communicate with each other enabling the device to operate. The main systems are:

- Physical assembly: comprising the culture chamber, mounting stand and instrumentation box.
- Operating software: comprising the mechanical load controller, the data acquisition module, the stimulator controller and the temperature regulator.
- Hardware: comprising the V273 linear actuator (Physik Instrumente, Cranfield, Wharley End, UK), F30 force sensor (Harvard Apparatus, Holliston, MA, USA), RaspberryPI, custom PCB (printed circuit board), custom. stimulator, custom temperature controller, NI USB‐6001 data acquisition (DAQ) device (National Instruments, Austin, TX, USA), custom force sensor amplifier and heating pads.
- Data analysis software: comprising the data visualisation and analysis software.

In the following sections, these parts are examined. Where relevant, the rationale behind specific technical decisions and their link to biological experiments is provided.

### 2.3 MyoLoop physical assembly

Physical assembly parts were rendered in Fusion360 (Autodesk, San Francisco, California). The components were then machined in the Imperial College London Mechanical Instrumentation Workshop.

#### 2.3.1 Culture chamber and mounting stand

**FIGURE 3:**
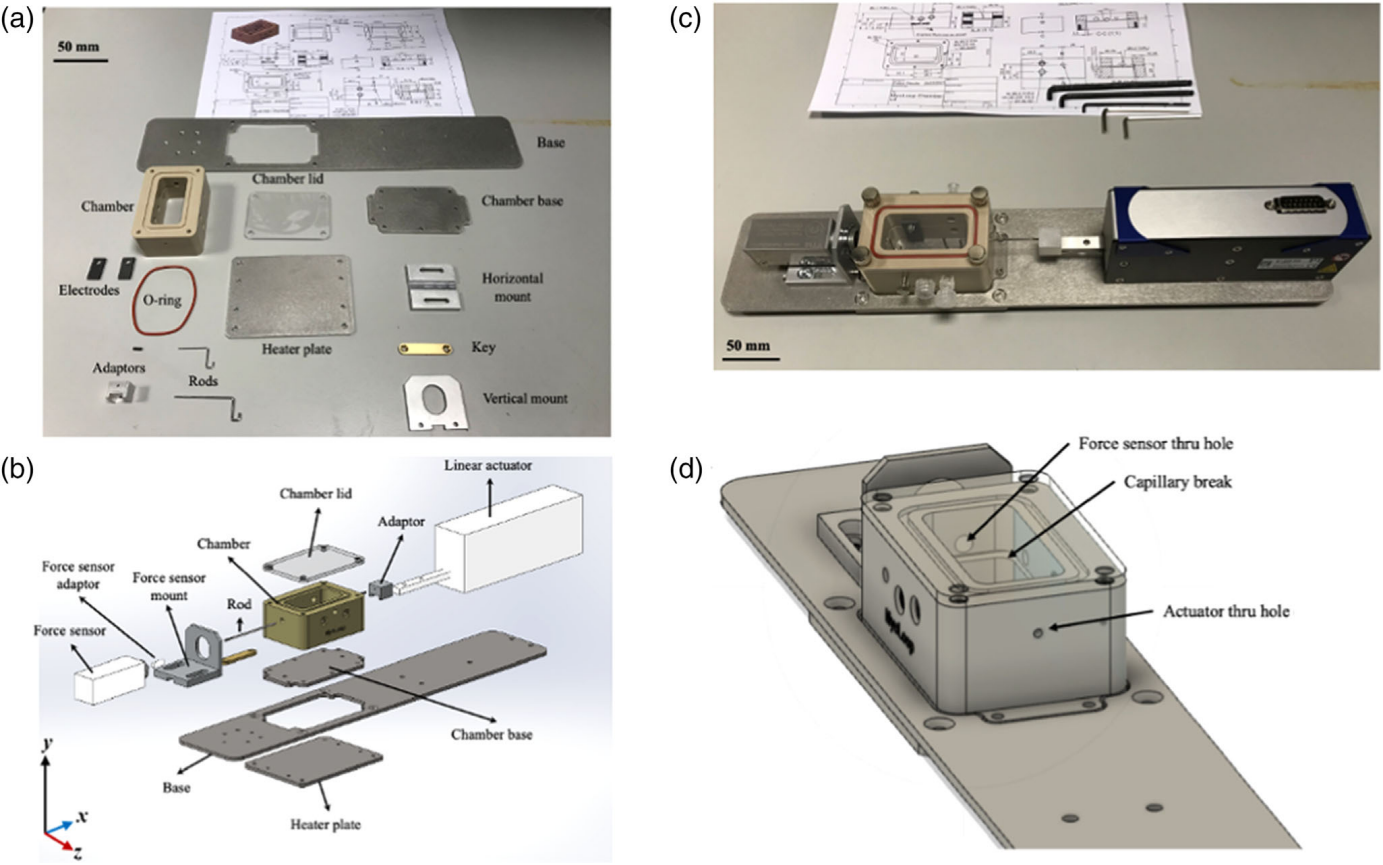
MyoLoop physical assembly. (a) MyoLoop physical assembly components. All components were designed in Fusion360 and subsequently manufactured at Imperial Instrumentation Workshop. (b) Exploded view of all components. (c) Assembled MyoLoop. (d) Close‐up view of chamber in 3D.

The physical assembly consists of the culture chamber and mounting stand (Figure 3). The culture chamber harbours a single LMS during experiments. The chamber is detachable from the rest of the physical assembly enabling easy sterilisation. To maintain the contents of the culture chamber at physiological temperature the chamber floor was made from 316 stainless steel. On the underside of the chamber floor, a silicone heating pad was attached and controlled by the MyoLoop software enabling heating of the medium inside the culture chamber by means of convection.

#### 2.3.2 Instrumentation box and hardware

**FIGURE 4:**
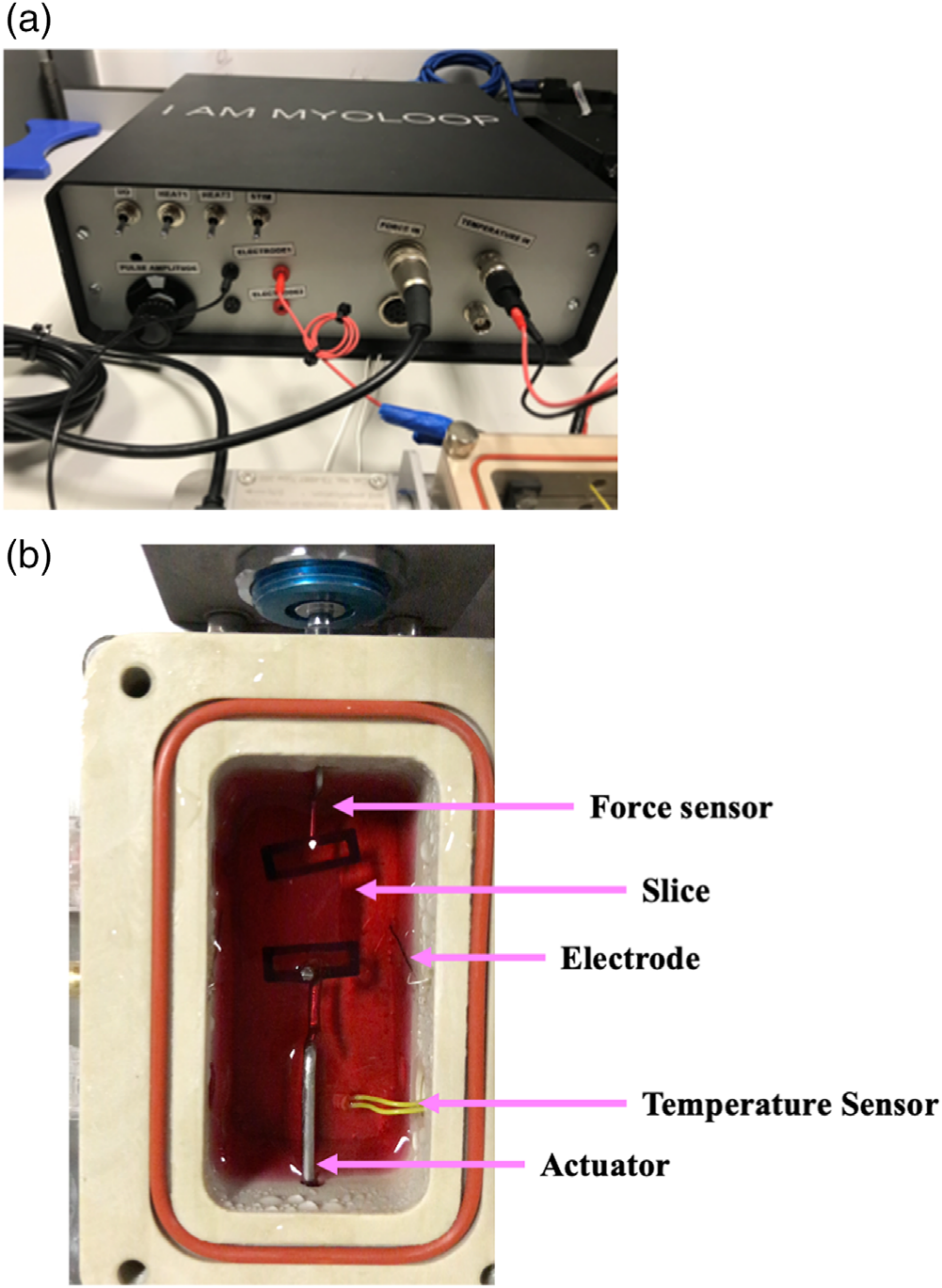
Instrumentation box and PCB. (a) MyoLoop instrumentation box. (b) MyoLoop chamber top‐down view. Notice slice mounted between actuator and force transducer, platinum electrodes and temperature sensor.

All MyoLoop electronics were enclosed within an aluminium instrument case (Figure 4). The front and back panel dial positions and drill sizes were designed on Fusion360, and CNC machined on the panels provided with the box. Dials and enclosure box were purchased from RS Components, Corby, UK.

The instrumentation box consisted of the MyoLoop hardware. This included the custom PCB, the NI USB‐6001 data acquisition device, as well as the RaspberryPI. Together, these components enabled sampling of the force produced by the LMS and actuation of a length waveform on the tissue leading to the recreation of force–length loops.

#### 2.3.3 Force sensor amplifier

LMS force generation was acquired by the F30 force transducer (Harvard Apparatus). An amplifier was developed for this sensor based on the INA125 instrumentation amplifier (Texas Instruments, Dallas, TX, USA). The gain of the amplifier was set based on the following equation according to the INA125 technical datasheet: $\mathrm{Gain}\;=\;4+\frac{60\;k\Omega }{R_{g}}$, with *R*_g_ being the resistance controlling the amplification gain. To calculate the required gain, the F30 specifications were used together with the sensitivity range of the USB‐6001 DAQ device.

#### 2.3.4 Stimulator and temperature controller

The temperature of the medium in the culture chamber was kept within physiological range by the temperature controller unit. This comprised a rapid response time 10 kΩ negative temperature coefficient thermistor (TE Connectivity, Schaffhausen, Switzerland), a 7.5 W silicone heating pad (RS Components), and an analog‐to‐digital converter (MCP3008, Microchip Technologies, Chandler, AZ, USA) connected to a RaspberryPI, and controlled by custom python codes. Continuous fluid recirculation by means of a peristaltic pump prevented temperature gradients in the culture chamber.

**FIGURE 5:**
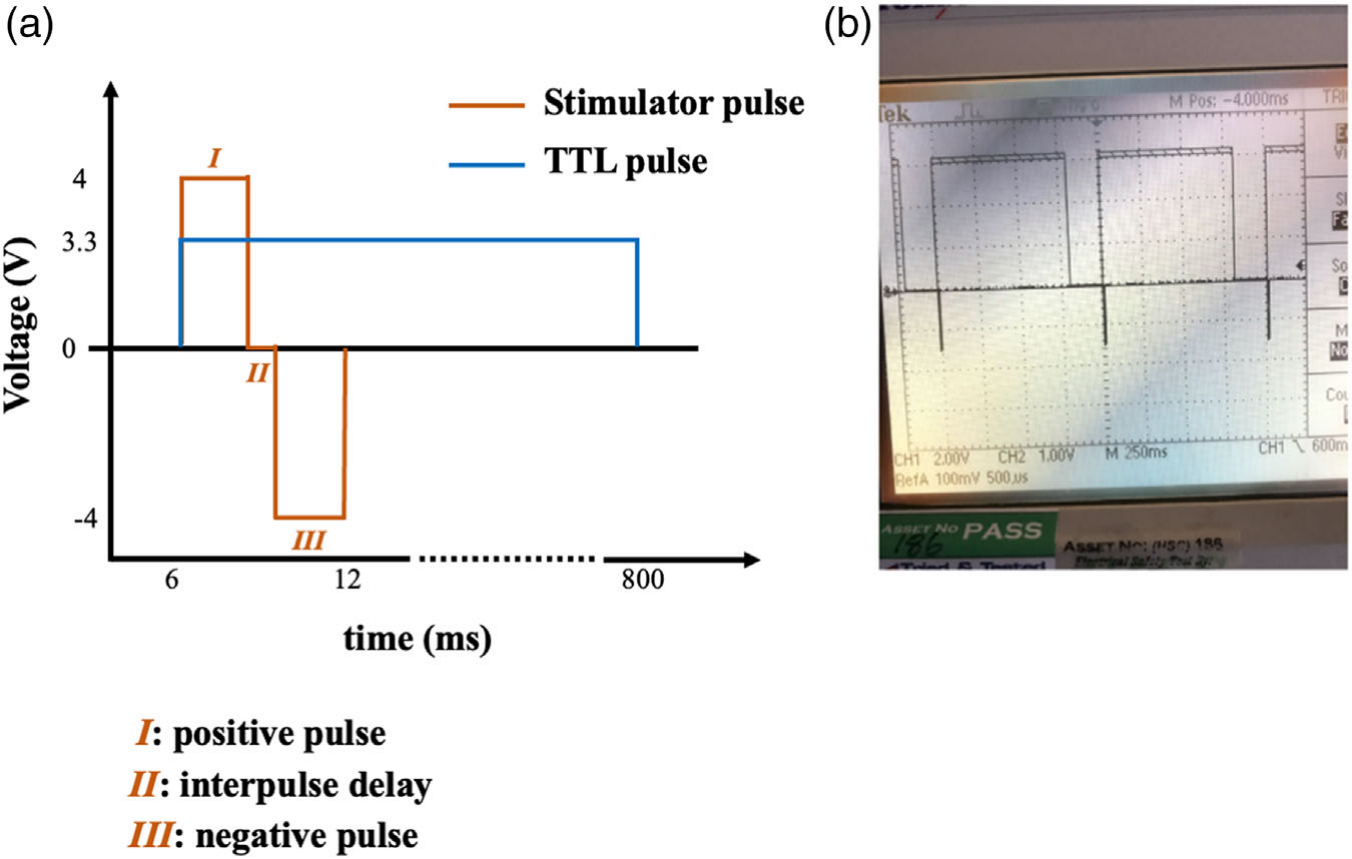
Biphasic stimulation pulse and TTL signal. (a) Diagram showing the biphasic pulse from LT1970 custom stimulator used to depolarise the LMS, as well as the TTL pulse generated by the RaspberryPI to control the actuator. The two pulses overlap enabling electromechanical coupling. (b) 800 ms ON and 200 ms OFF 1 Hz TTL pulses used to control the actuator. Frequency of TTL pulse is adjustable from user interface enabling different frequencies to be used for LMS pacing. Also observe negative pulse at the beginning of every TTL signal, corresponding to the stimulation pulse.

The stimulator controller unit delivered biphasic pulses that paced the LMS and set the TTL (Transistor‐transistor logic) triggers for actuator movement and NI DAQ (Figure 5). The LT1970 chip power‐op amplifier was used for this with timing controlled by custom RaspberryPI codes. A graphical user interface prompt enabled the experimenter to select both the desired temperature and the stimulation pulse width and frequency (it can be seen in Figure 2). The stimulation current was set using the rotatory dial on the MyoLoop box and provided ∼50 mA of current. Different stimulation frequencies are possible and programmable by the MyoLoop GUI (Figure 2). LMSs were field stimulated using the platinum electrodes on either side of the culture chamber.

### 2.4 MyoLoop software and hardware

#### 2.4.1 Data acquisition and work loops

**FIGURE 6:**
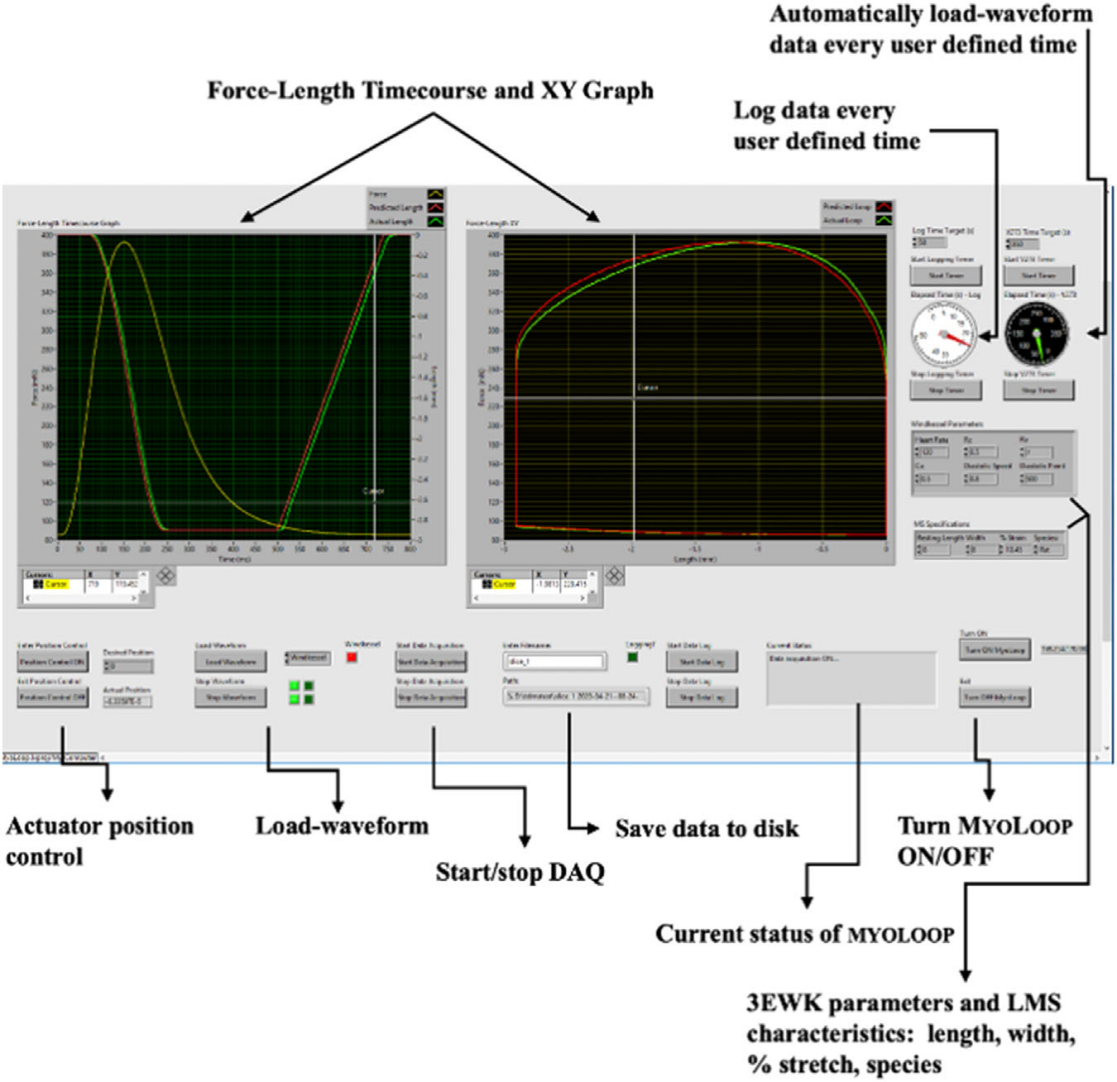
MyoLoop main user interface. Two main real‐time display panels make up the MyoLoop user interface. Relevant control buttons are found on the right‐ and bottom‐side of the GUI. The command buttons are annotated here. The panel on the left displays the force generated by a LMS as well as the length waveform being actuated on the LMS in real time. The panel on the right displays the force–length loop of the corresponding data. The user interface allows the user to record the data either continuously or every user‐defined interval (e.g., every 5 min), change 3EWK model parameters (Ra, Ca, Rc), as well as LMS characteristics (width, length, species). All data are saved to a user‐specified location and can be subsequently analysed offline using custom software.

All data acquisition software was coded in LabVIEW (National Instruments) using a queued message handler design. Software was written to solve the 3EWK model, and apply mechanical load based on the output of the model to the LMS using the linear actuator, acquire the force generated by the LMS using the force sensor, display the force–length loops in real time, save the data, as well as all relevant user‐interface commands. The LabVIEW user interface is shown in Figure 6.

**FIGURE 7:**
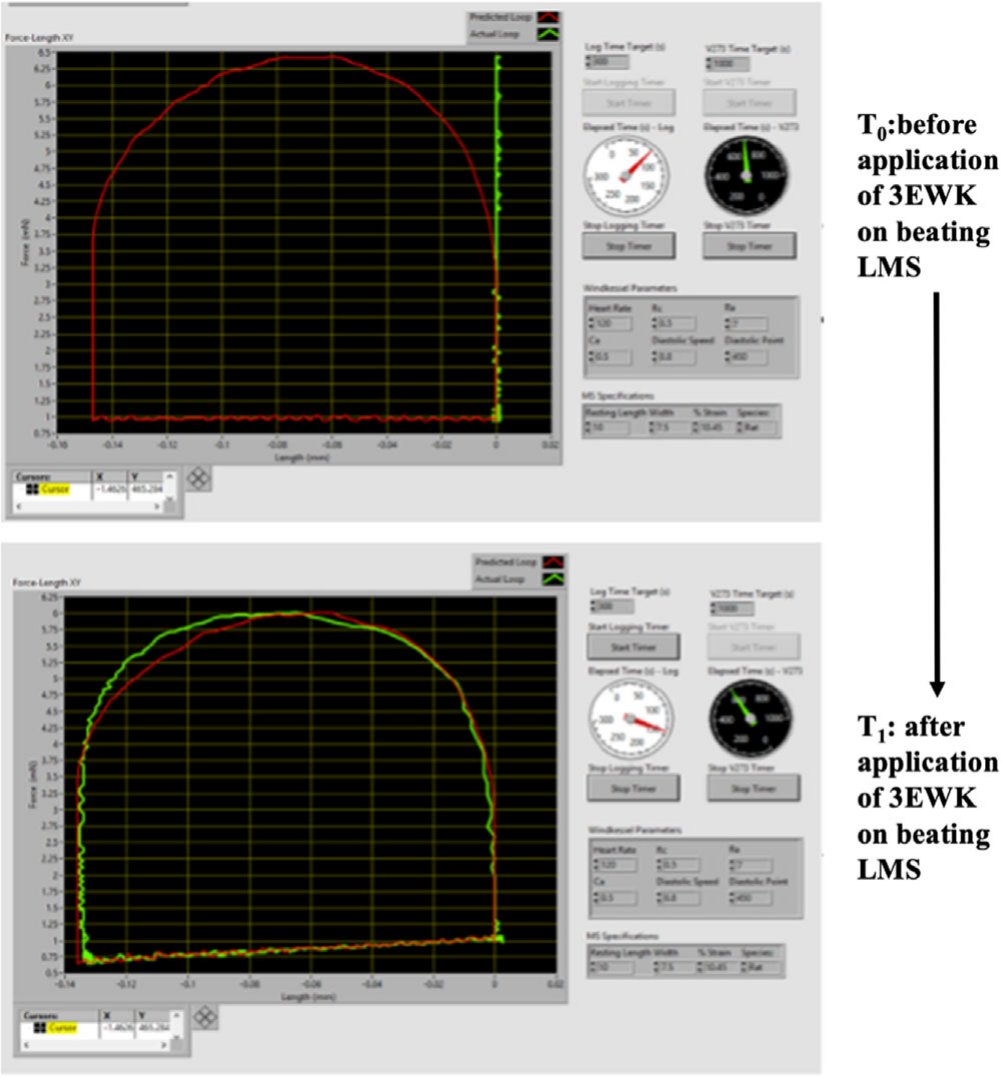
Application of mechanical load in beating LMS doing a work‐loop. Top, LMS beating in MyoLoop under isometric load (green line), the default setting in MyoLoop. When desired by the user, the isometric load mode be switched to a 3EWK mode whereby the LMS is looping. The red line demonstrates the output of the 3EWK based on the force being generated by the LMS on the previous beat. Bottom, application of the predicted length waveform on the beating LMS on the next beat—this corresponds to the 3EWK mode of MyoLoop. Notice that the length waveform being actuated on LMS (green) converges with the predicted waveform (red).

The algorithm by which the 3EWK model was used to apply mechanical load to the LMS has been previously described in Pitoulis et al. (2021). Briefly, the 3EWK model controlled the LMS rate and amount of shortening during a muscle twitch. To do that, the force generated by a beating LMS was first sampled at 1 kHz. The force data were then converted to pressure using Laplace's law of the heart (Grossman et al., 1975) and fed to the numerically solved 3EWK model. The model outputted a corresponding volume waveform, which was then converted to length based on the LMS dimensions and a spherical model of the heart (De Tombe & Little, 1994). On the next beat, the length waveform was applied to the LMS in sync with the depolarising pulse from the stimulator. This enabled the length waveform to be synchronised with the contracting LMS. The diastolic phase was controlled by a linear ramp which stretched the LMS from end‐systolic length back to the user‐defined preload (end‐diastolic length). This whole sequence was visualised in real time in the user interface (Figure 7).

### 2.5 Wet experiments

#### 2.5.1 Preparation of LMSs

LMSs were prepared from adult Sprague–Dawley rats as previously described in Pitoulis et al. (2021). Briefly, adult male Sprague–Dawley rats were killed under isoflurane anaesthesia by cervical dislocation and carotid artery dissection. The heart was rapidly extracted and first placed for 5 s in a 60 mL vial containing 37°C modified heparinized (1000 IU/mL) Tyrode's solution (30.0 mM 2,3–butanedione monoxime, 9.0 mM KCl and 1.0 mM CaCl₂ at pH 7.40), and then in a 60 mL vial containing 4°C modified heparinized solution (as above). The left ventricular free wall was then dissected from the remainder of the heart using forceps, microscissors and a scalpel, and was made into a flat left ventricular block. This was glued epicardium face down using histoacryl surgical glue (Braun, Kronberg, Germany) to a 20 × 20 × 3 mm block of agarose (4% w/v) (Sigma‐Aldrich (Merck Life Science UK Ltd, Gillingham, UK)) and mounted to a specimen holder, again using histoacryl surgical glue. The specimen holder containing the ventricular block was then mounted to the vibratome (7000smz2, Campden Instruments Ltd, Leicester, UK) and submerged in oxygenated (100% medical grade O₂; BOC, Surrey, UK) 4°C modified Tyrode's solution. LMSs were then generated using a 0.03 mm/s vibratome advance speed, 80 Hz frequency and 2 mm amplitude. They were examined under ×10 magnification light microscopy to determine myocardial fibre orientation and cut using a blade parallel to fibre direction to form roughly 10 mm × 8 mm × 300 μm‐thick LMSs. Final length and width of LMSs were recorded using digital callipers at LMS slack length (RS Components), and custom 3D printed polylactic acid (RS Components) holders of rectangular shape were glued to each end of the LMS perpendicular to fibre direction using surgical glue.

#### 2.5.2 Acute force–length loops

**FIGURE 8:**
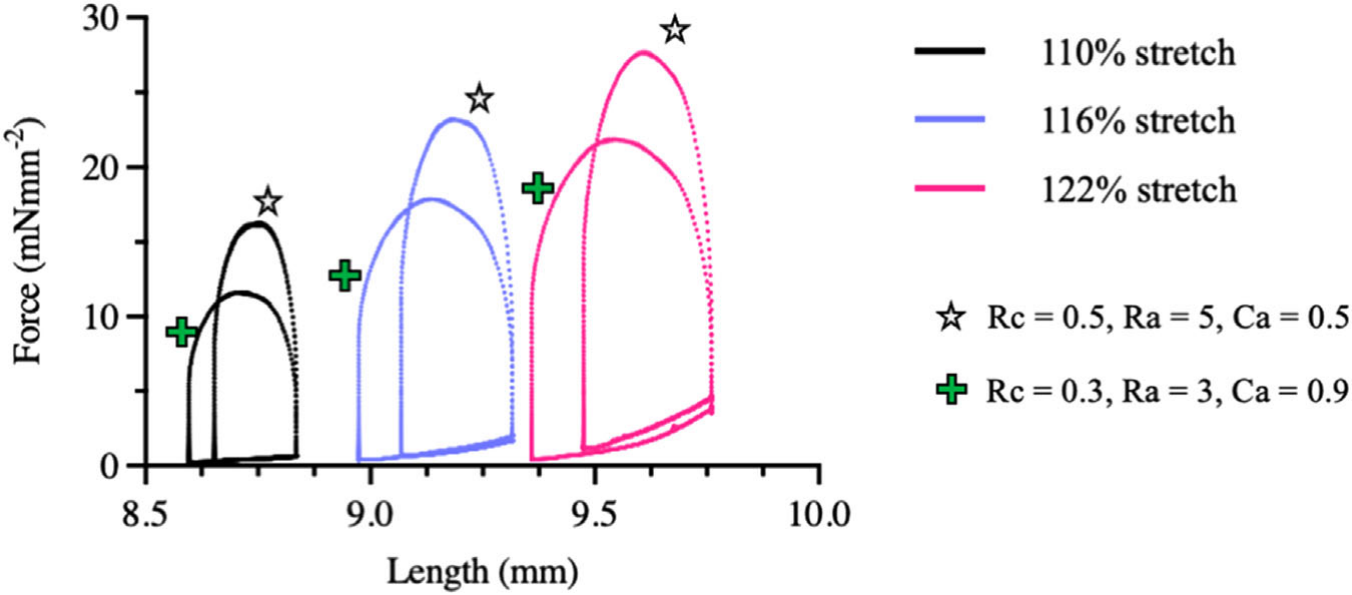
Acute force–length work loops. A freshly prepared rat LMS was mounted on MyoLoop and allowed to perform work‐loops using the 3EWK at three different preloads (stretch of 110%, 116% and 122%, and equivalent to 2.09, 2.17 and 2.25 μm in SL), and two different afterloads: (a) (Rc = 0.5, Ra = 5, Ca = 0.5 and (b) Rc = 0.3, Ra = 3, Ca = 0.9). These experiments demonstrate the ability of MyoLoop to control both preload and afterload.

To validate the ability of MyoLoop to perform work‐loops, freshly prepared rat LMSs were stretched to three different preloads corresponding to percentage stretches of 110%, 116% and 122% whilst inside the device, corresponding to SL of 2.09, 2.17 and 2.25 μm. Then, at each diastolic length, the LMSs were switched from the isometric mode to the 3EWK and allowed to loop using two different 3EWK parameters (Figure 8). These were (a) Rc = 0.5, Ra = 5, Ca = 0.5 and (b) Rc = 0.3, Ra = 3, Ca = 0.9. These values are arbitrary and relative to each other, and were chosen in order to simulate conditions of low and high afterload respectively. Examples of relevant 3EWK parameters for mechanical load manipulation are also provided in Table 1 below.

**TABLE 1:** Examples of 3EWK and preload parameters for simulation of different mechanical loads using MyoLoop.

Simulated condition	Preload (μm)	Rc	Ra	Ca
Pressure overload (e.g., aortic stenosis)	2.1	1	10	0.5
Volume overload (e.g., mitral regurgitation)	2.3	0.5	5	0.5
Aorta non‐compliance (e.g., age‐related aortic stiffness)	2.1	0.5	5	0.2

#### 2.5.3 Culture of LMSs in MyoLoop

Rat LMSs were cultured in MyoLoop for 72 h. To do that, freshly prepared LMS were mounted between the MyoLoop actuator and the force sensor using forceps. MyoLoop was turned on and the LMS was progressively stretched using the LabVIEW GUI until the LMS muscle length was equivalent to a SL of 2.1 μm. This was accomplished by using a linear relationship between LMS resting length and SL obtained previously from laser diffraction studies (Watson et al., 2019), which provide an estimate value of the mean SL across cardiomyocytes in the preparation. The stimulator was then turned on, and the 3EWK mode initiated.

As our intention was to validate the ability of MyoLoop to culture LMS and reproduce the results obtained previously in the commercially available modified systems (Pitoulis et al., 2021), the afterload and preload parameters were the same as those used in Pitoulis et al. (2021). As such, these corresponded to physiological preload (2.1 μm) and afterload (Ra = 7, Rc = 0.5, Ca = 0.5), with a 200 ms diastolic ramp phase after isometric relaxation, which brought the LMS back to end‐diastolic length.

Medium‐199 with Earle's salts (Sigma‐Aldrich) was used as culture medium. This was supplemented with 3% penicillin–streptomycin, 1:1000 insulin–transferrin–selenium, and 20 μg/mL l‐ascorbic Acid (all from Sigma‐Aldrich) as well as 4 nM (–)‐adrenaline (+)‐bitartrate salt, 2.5 nM l‐(‐)‐noradrenaline (+)–bitartrate salt, 2.15 nM 3,3′,5‐triiodo‐l‐thyronine and 100 nM dexamethasone as previously described (Pitoulis et al., 2021). This medium has been optimised and previously used by us to culture LMS (Pitoulis et al., 2021). Medium was recirculated using an external MasterFlex peristaltic and 3/8 inches platinum‐cured peristaltic tubing (Cole‐Parmer, Saint Neots, UK) with inflow/outflow rates equal to 50 mL/min and connected to a 500 mL external medium bottle using a double perfusion system gassed with 95% O_2_–5% CO_2_. Fresh medium (slowly heated to 36°C) was added to the external medium bottle every 24 h and old medium discarded. The temperature of the chamber was kept at 36°C, and the LMS stimulated at 1 Hz and 6–12 ms pulse width using the MyoLoop GUI.

The data acquisition system displayed all beats throughout the 72 h culture in real‐time but only one beat every 5 min was saved. This was done for data storage purposes.

### 2.6 Analysis

#### 2.6.1 Data analysis

Force and length data were continuously monitored on MyoLoop and saved in .tdms format by default. A LabVIEW .tdms reader was coded, which allowed visualisation of data as well as conversion of .tdms files to .txt or .csv for further analysis. Passive and active tension generated by the LMS can be obtained from the force–length loops by analysing the force component alone. Likewise, stroke length and length‐waveform characteristics can be obtained by analysing the length component of the data.

#### 2.6.2 Statistics

Statistical analysis was performed in Prism 8 (GraphPad Software, San Diego, CA, USA). First, a D'Agostino–Pearson and Shapiro–Wilk test was used for assessment of normality. Two‐way analysis of variance (ANOVA) with Tukey's *post hoc* test was used for determination of statistical differences between group means (active and passive force throughout culture).

## 3 RESULTS

Here, we have demonstrated the development of MyoLoop, a bioreactor capable of simulating the cardiac cycle on LMS using a 3EWK model and designed specifically for long‐term culture experiments. Both preload and afterload are parameterised and easily manipulated in MyoLoop (Figure 8), allowing for the desired mechanical load profile, physiological or pathological, to be applied to LMSs.

**FIGURE 9:**
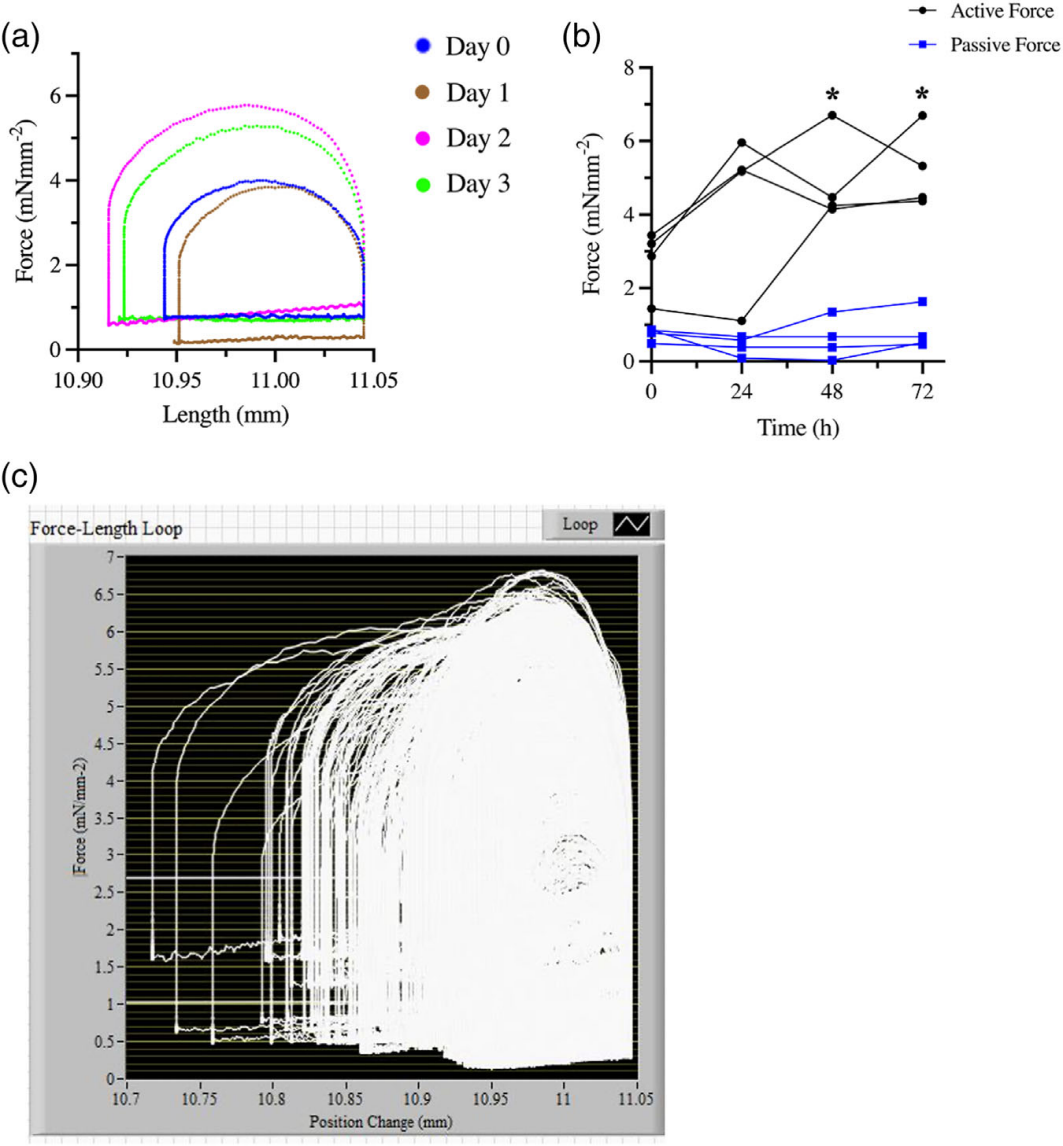
Culture of rat LMSs for 72 h on MyoLoop under physiological load. (a) Force–length loops of rat LMS in MyoLoop corresponding to day 0, 1, 2 and 3. (b) Active and passive force of LMSs during 72 h culture. Both the temporal pattern of and the active and passive force of LMSs in MyoLoop were similar to those performed by LMSs in our previous set‐up in the lab (Pitoulis et al., 2021) (*n* = 4, mean ± SEM). (c) Overlay of all force loops performed over 72 h culture by a single LMS. A force–length loop was acquired every 5 min throughout the culture. Notice that the LMS work loops vary throughout the culture. This reflects the feedback algorithm which is used by MyoLoop whereby the length‐waveform performed by the LMS is not constant, but rather a function of the force that the LMS is producing. *Statistically significant difference between active tension in day 0 versus day 3 and day 4.

We show that MyoLoop can be used for chronic culture of LMS under conditions of continuous electromechanical stimulation, mimicking the cardiac cycle (Figure 9), and demonstrate that under these conditions, LMSs can be maintained in culture for at least 3 days. The preload and afterload parameters used for this in vitro culture were the same as those used in Pitoulis et al. (2021) and we show similar results to those obtained previously.

## 4 DISCUSSION

### 4.1 Overview of MyoLoop and comparison with other culture platforms

In an effort to continuously approach physiological relevance, many different LMS culture platforms have been developed by us and others (Fischer et al., 2019; Ou et al., 2019; Pitoulis et al., 2021; Qiao et al., 2018; Watson et al., 2019; Miller et al., 2022). Watson et al. (2019) developed culture chambers with stretching devices capable of culturing up to six LMSs simultaneously under isometric load. Using this approach, rat LMSs cultured at different preloads over 24 h resulted in different structural and functional remodelling (Watson et al., 2019). In similar fashion, Fischer et al. (2019) developed a standalone device that could auxotonically load up to eight LMSs. This device also afforded higher throughput and continuous monitoring of force generated by LMSs during culture. Remarkably, LMSs from human samples were cultured for up to 4 months in this platform. More recently, Miller et al. (2022), designed a culture system which utilised a pneumatic device to adjust preload. LMSs were cultured for up to 12 days with minimal differences in transcriptional profile when comparing fresh and cultured LMSs. However, fine control of afterload or preload was not demonstrated in this system, and tissue contractility and kinetics are assessed crudely using a camera set‐up.

Most importantly, none of these approaches captured the complexity of the cardiac cycle and its distinct phases. Recently, and before the development of MyoLoop, we had shown that (a) pressure–volume loops could be actuated on LMSs as force–length loops using a 3EWK model, and that (b) LMSs could be cultured under this constant dynamic mechanical load during culture (i.e., ‘looping’) (Pitoulis et al., 2021). However, to do that we had to extensively customise commercially available equipment, which was not suitable for the culture of cardiac tissue. This was expensive and non‐scalable. The initial difficulties we encountered with modifying such equipment led to the development of MyoLoop, a novel stand‐alone bench‐top device for electromechanical stimulation of cardiac tissue in vitro.

Compared with to previous platforms that were developed to perform work‐loops on LMSs (Pitoulis et al., 2021) or culture LMSs under isometric load (Watson et al., 2019), MyoLoop offers a number of improvements. First, except for a peristaltic pump, which recirculated the culture medium, MyoLoop does not require any other third‐party equipment to operate. It has an integrated temperature control unit, stimulator, data acquisition system, as well as real‐time visualisation and data analysis software. By minimising the use of third‐party equipment, we were able to (a) significantly cut the costs to produce the device, (b) make them customisable, (c) optimise components, specifically culture, as well as (d) develop the device to be fully autonomous. In contrast, other systems including that developed by us (Pitoulis et al., 2021; Watson et al., 2019) and others (Ou et al., 2019) required the use of incubators, stimulators, peristaltic pump and DAQ systems for subsequent contractility measurements, while that of Fischer et al. (2019) and Qiao et al. (2018) required an incubator as well as DAQ systems for contractility assays.

Furthermore, MyoLoop is unique in that it parameterises both preload and afterload (Figure 8) allowing for fine control of mechanical load during in vitro experiments. In its default state, MyoLoop permits users to choose between either an isometric loading protocol or a 3EWK model; however, the mechanical load controller is customisable using a few lines of code accessible to the user. Any user‐defined mathematical function can be inputted on the MyoLoop back‐end, such as an auxotonic model of contraction or a four‐element Windkessel model.

MyoLoop was optimised specifically for chronic culture experiments, and as such has several features to enable that including being modular and able to maintain sterility, constant looping of LMSs, and continuous or timed acquisition of data. We show that by using MyoLoop we are able to maintain rat LMSs for up to 3 days with similar results to those acquired previously in Pitoulis et al. (2021), reflected both by the passive and active tension generated by slices during the culture experiments as well as by the work loops morphology. Furthermore, the MyoLoop data demonstrated the typical temporal pattern in LMS contractility characterised by an initial decrease, followed by an increase in LMS force production consistent with previous publications from our lab (Pitoulis et al., 2021) and others (Fischer et al., 2019).

### 4.2 Future experiments and MyoLoop potential

The ability to culture LMSs in vitro offers many avenues for future cardiovascular research. For example, we previously used the 3EWK model to culture LMSs under either volume‐ or pressure‐overloaded conditions and found that LMSs remodel differentially based on mechanical load profile applied to them (Pitoulis et al., 2021). Future experiments can further expand on these results and identify responsible pathways.

As preload and afterload can be changed not only at the beginning of culture but also during culture in MyoLoop, experiments could focus on reverse remodelling and mechanical unloading. For example, LMSs could first be overloaded by using the 3EWK to model pressure overload over 48 h and then unloaded over the next days to study whether pathological phenotypes can be reverted and, if so, the mechanisms responsible for this. In addition, as the mechanical load controller is customisable, the effects of mechanical loading conditions that simulate unloading interventions, such as left ventricular assist devices, could be studied in our platform.

### 4.3 Limitations

One of the main limitations of MyoLoop is throughput. Although the device was designed to be scalable based on cost, at present only one LMS is run per culture chamber, and two seperate culture chambers can be accomodated per MyoLoop system. Expansion to multiple parallel systems is necessary for throughput. This is in contrast to other systems such as the standard culture chamber of Watson et al. (2019) and the culture platform of Fischer et al. (2019), which can accommodate four and eight LMSs, respectively.

Although MyoLoop was designed to be user‐friendly, it is a more complex system than the culture chambers with stretchers by Watson et al. (2019) or the system designed by Fischer et al. (2019), as an understanding of the 3EWK model is necessary to study physiology reliably. It is also important to mention that the actuator movement performed by MyoLoop based on the 3EWK model only simulates shortening across the longitudinal plane and does not simulate the helical ‘squeeze’ contraction movement of the heart in vivo. Finally, although the device is largely self‐contained, a peristaltic pump is necessary to recirculate the culture medium; future iterations of the device will incorporate a mechanism to stir the medium and eliminate the need for third‐party components.

### 4.4 Conclusion

Translational cardiovascular research is limited by the lack of physiological relevance of in vitro cardiac models. Failure to approximate the electromechanical events of the cardiac cycle is a key reason for this. At present, there are no commercially available culture systems that can (a) recreate the cardiac cycle and (b) reliably culture cardiac tissue without the need for extensive modifications. We believe pathophysiologically relevant mechanical loading of cardiac tissue will be essential for the future of cardiac translational research, and we developed MyoLoop specifically for this – a bioreactor that can keep LMSs and other cardiac preparations ‘looping’ during in vitro culture.

## AUTHOR CONTRIBUTIONS

Fotios G. Pitoulis conceptualised, designed, coded and developed the device including electrical circuits, 3D renderings, software, all algorithms of MyoLoop, run the experiments and wrote the manuscript. Jacob Smith edited the manuscript. Blanca Pamias‐Lopez wrote and edited the manuscript. Danika Hayman wrote and edited the manuscript. Pieter P. de Tombe conceptualised designed, coded and developed MyoLoop, and edited the manuscript. Cesare M. Terracciano conceptualised, designed, developed MyoLoop and edited the manuscript. All authors have read and approved the final version of this manuscript and agree to be accountable for all aspects of the work in ensuring that questions related to the accuracy or integrity of any part of the work are appropriately investigated and resolved. All persons designated as authors qualify for authorship, and all those who qualify for authorship are listed.

## CONFLICT OF INTEREST

Fotios G. Pitoulis, Pieter P. de Tombe and Cesare M. Terracciano are inventors of a patent (Pitoulis et al., 2019) protecting the IP generated during Fotios's MD‐PhD (Reference number: 10482) for MyoLoop. The IP is owned by Imperial College London.

## Data Availability

All the data from the wet lab experiments conducted herein is available in a public repository. The link will be provided upon reasonable request.
